# A novel image encryption technique using hybrid method of discrete dynamical chaotic maps and Brownian motion

**DOI:** 10.1371/journal.pone.0225031

**Published:** 2019-12-19

**Authors:** Majid Khan, Fawad Masood, Abdullah Alghafis, Muhammad Amin, Syeda Iram Batool Naqvi

**Affiliations:** 1 Cyber and Information Security Lab (CISL), Institute of Space Technology, Islamabad,Pakistan; 2 Department of Applied Mathematics and Statistics, Institute of Space Technology, Islamabad, Pakistan; 3 Department of Electrical Engineering, Institute of Space Technology, Islamabad,Pakistan; 4 King Abdulaziz City for Science and Technology Riyadh, Saudi Arabia; 5 Department of Avionics, Institute of Space Technology Islamabad, Pakistan; Nanjing University of Information Science and Technology, CHINA

## Abstract

Information security is an important and critical subject of the current digital era. Nowadays, almost all information is digital in nature and security from hackers and eavesdroppers has become vital in civil (big giant corporations) as well as in defense organizations. One type of information in bit streams is in the form of digital images. In this article, an idea to combine Brownian motion with ternary unique orientation has been implemented which is related to random motion over time and spatial coordinates. Moreover, chaotic dynamical map has been used to add one more security level to the proposed encryption scheme. The proposed scheme is evaluated on different statistical tests and these results are compared with already existing benchmarks. The results show that the proposed algorithm has better security performances as compared to existing image enciphering schemes.

## 1. Introduction

The security of digital information in wireless and wired communication media such as Wi-Fi and Ethernet is one of challenging issues of the contemporary world. Today, advancement in science and technology makes our lives easier as compared to earlier decades and the history is evident that this process of bringing comfort and ease pervades all aspect of human lives. The idea of transforming any analogue information for instance text, image, audio and video into digital bitstreams is one of the grounding breaking discovery by Claude Shannon in 1948. Shannon put forth the idea that once information becomes digital, it could be transmitted without error. This revolutionary concept led directly to digital storage media such as hard disks, CDs and USBs. The privacy of information and copyright protection became critical issue of the digital world. The eavesdropping of the digital information is possible in any part of the communication system from source through the transmitting medium to the sink. These cyber threats of data eavesdropping are developing at fast pace. Data communication has become pervasive in society spanning individuals, groups and state institutions. Many applications like video conferencing, photo album, medical images and databases require quick, reliable, computationally less intensive and robust systems for secure transmission of digital information from one end to another end. Therefore, privacy of data is a crucial factor which must be first choice to looked into to provide secure communication. In video and audio communication real time high data transmission rates at one hand provides seamless communication but on the other hand it requires robust security against interception and eavesdropping and extraction of plain text information. The major principles of cryptography are confidentiality, integrity and authentication that ensure secure communication. The confidentiality of information can be achieved through encryption procedure. The earlier cryptosystem used either substitution or permutation but separately and independently which means these schemes did not utilize confusion and diffusion simultaneously. The separate usage of confusion and diffusion in earlier schemes was vulnerable to different cryptographic attacks. Claude Shannon in 1949, devised a mechanism of confusion and diffusion in order to design secure cryptosystems. The modern information security system named it substitution-permutation network (SP-network). Traditional symmetric ciphers such as advanced encryption standard (AES) and data encryption standard (DES) and all other variants of these cryptosystems are designed with good confusion and diffusion properties [1–11].

Several encryption algorithms were designed for the confidentiality of digital information which utilized SP-network. A large number of modern ciphers schemes use many aspects of chaos to enhance confusion and diffusion capabilities. The chaos theory is closely related with cryptography. The chaotic systems have characteristic similar to cryptography such as highly sensitive to chaotic parameters, initial conditions, its un-deterministic nature, pseudo-randomness and ergodicity etc., which are fundamental requirements while designing any modern cryptosystems [12–16]. The connection between chaos and cryptography introduced a new regime in modern information security. Therefore, chaos theory after 1990’s, was extensively utilized for developing robust cryptosystems [17–70]. Our idea here is to use two dimensional chaotic maps along with Brownian motion in order to design a new scheme for the confidentiality of digital images.

Brownian motion is random (zig zag) movement of particles that are suspended in fluid (liquid or gas) resulting due to fast collision of liquid or gas molecules. The term ‘Brownian motion’ is here mathematical term or model use for designing of secure cryptosystem and is use to describe ‘random movement of particles’. In 2014, Wang and Xu [64] considered single particle of Brownian theory as a pixel of image and used Monte Carlo method and encrypt original test image. In 2015, Zhu [65] had cracked the algorithm designed by Wang *et al* [64] because the system designed was based on permutation and diffusion sequence by Wang *et al* [64] that was not related to plaintext image thus the method designed by Wang *et al* [64] was unfeasible to show resistivity against the chosen plain text.

The rest of the paper is organized as follows: Section 3 briefly introduces basic preliminaries used in designing of strong cryptosystem. The succeeding section 4 describes the proposed algorithm. The simulation results and security analysis are shown in section 5 and finally, we summarized our conclusion and results in section 6.

## 2. Motivation

Information transmission through open public network are vulnerable to assorted type of attacks so designing these hybrid systems emphasized to meet security deficiency for real time multimedia communication. The proposed system is based on Brownian motion of particles along three different directions of *X*, *Y* and *Z* axis and inclusion of certain discrete time chaotic maps is to enhance its security to some more steps. The system helped us in transforming plain information to intelligible form. In first phase Brownian motion of particles are initialized and then stored its position at each iteration. The unique ternary orientation provided some extra layer of security that increased number of conditions to encrypt original information in the form of digital image. The security provider may use single direction to secure their information as well as combined effect of unique three directions in spatial domain. This hybrid system based on chaotic behavior have some unique features i.e. highly sensitive in nature while altering its initial condition this system show highly randomness for different initial condition. The system has another unique feature of strange attractor i.e. for each small change in initial condition will give you complete different attractor which will create difficulty to intruder or advertiser to decipher original image. These properties motivated us to design this cryptosystem.

## 3. Some basic preliminaries

In this section, we have defined some basic definitions and concepts, which will be quite helpful for subsequent sections.

### 3.1 Chaotic systems

Chaotic systems have some properties like highly random in nature, strange attractor, aperiodicity, ergodicity and sensitive to its initial conditions which makes it suitable for designing secure cryptosystem [29]. If the value of the initial condition is insignificantly changed, the output of the system will show unexpected change. Many systems in nature around us exhibit chaos [67].

#### 3.1.1 Basin Chaotic Map

Chaotic maps have its intrinsic importance in designing secure cryptosystem. In order to make robust system, we introduced basin chaotic map and examined its desirable characteristics. It is discrete time two-dimensional chaotic map which takes some initial conditions e.g. *x*_0_ = −1.5, *y*_0_ = 1.5, and some other chaotic factors including *k*, *μ*, and *ε*. The mathematical expression for Basin chaotic map is defined as follow:

$$x_{n+1}=x_{n}+y_{n},$$ (1)

$$y_{n+1}=y_{n}+\varepsilon y_{n}+kx_{n}(x_{n}-1)+\mu x_{n}y_{n}.$$ (2)

#### 3.1.2 Ginger Breadman Chaotic Map

Ginger breadman is two-dimensional discrete time chaotic map which has noteworthy nature of randomness of chaos. The two-dimensional Ginger breadman chaotic map is represented in Eqs 5 and 6:

$$x_{n+1}=1-y_{n}+\left|x_{n}\right|,$$ (3)

$$y_{{}_{n+1}}=x_{n}.$$ (4)

### 3.2 Brownian Motion

**Fig 1:**
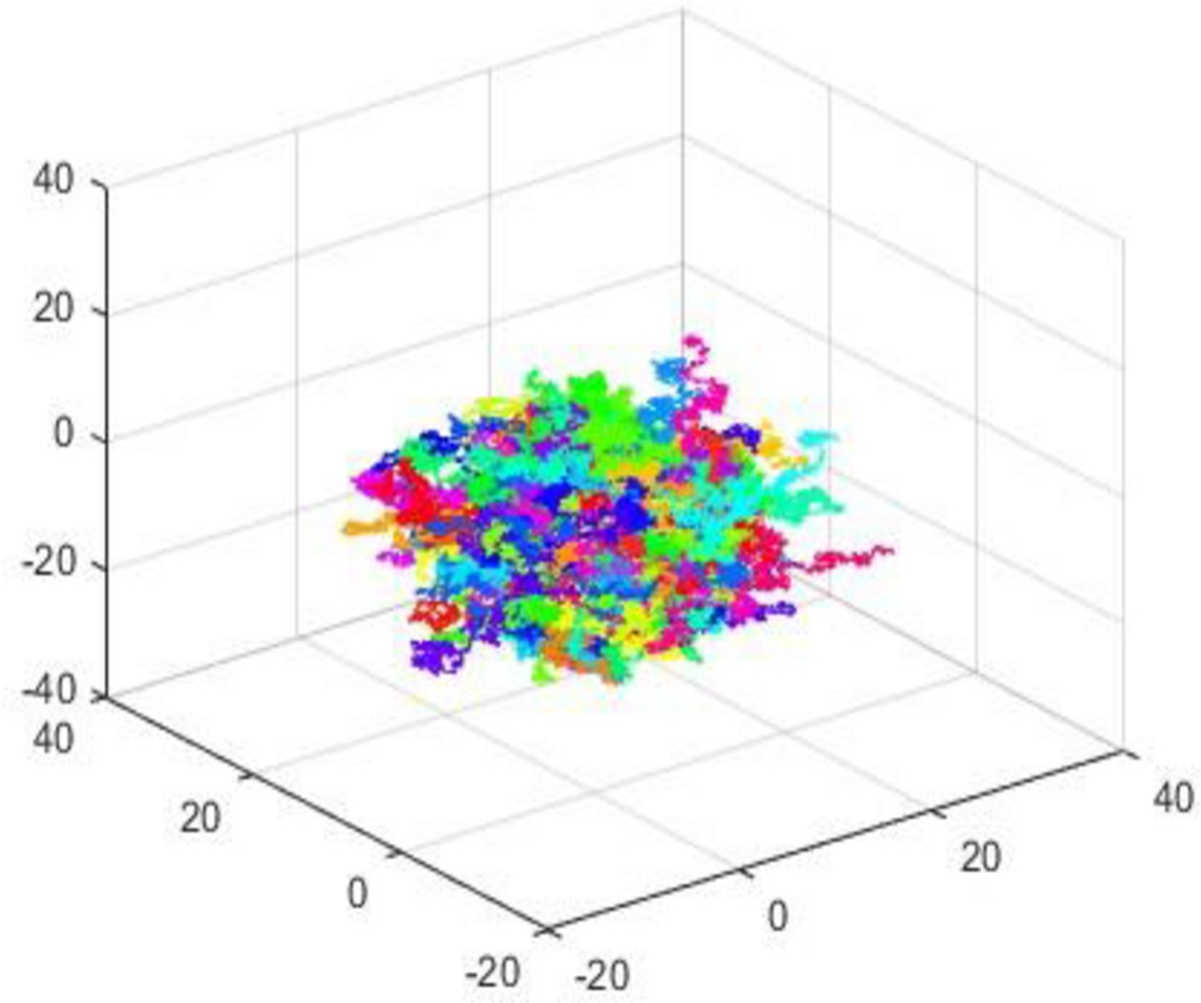
Brownian movement particles in ternary position.

It is random (zig zag) movement of particles along three different directions namely X, *Y* and *Z* axis (see Fig 1). The behavior of particles named after botanist Brown who worked on microscopic particles. The idea he revealed for the first time through pollen grains which fell into river and observed fluctuations and random motion in water though he didn’t find the reason of behavior he observed. Later one of the great scientist Albert Einstein published one of his article in 1905 that explained the exact random motion of particles which Brown had observed through motion of particles by water molecules and this was a very significant contribution to science. He concluded that the kinetic energy of molecules of water was responsible for the zig zag motion of the particles [30–31].

## 3.3 State of Art of Encryption for Proposed Scheme

Our main intention is designing robust algorithm having exceptional strength of resistivity against cryptanalysis and have negligible vulnerabilities to be cracked against gain of access of encrypted digital contents. In cryptanalysis where hacker study possible breaches and vulnerabilities in order to break down cryptographic algorithm. The robustness of proposed system can be validated using certain statistical tests that ensure the strength of proposed algorithm. The strength of cryptosystem highly depends upon two factors i.e. proposed algorithm for encryption and secrecy of keys used in proposed system to encrypt information. We used hybrid method of Brownian motion of particles and certain discrete dynamical chaotic maps to get maximum random sequence. We assumed and defined certain number of particles with respect to time before using certain chaotic maps. These particles helped us in achieving effect of zig zag motion of initially defined particles with respect to time and assumed number of particles. The assumed particles depend upon number of pixels in test image and the track changes due to changing effect of these particles. These particles are placed in spatial domain of three dimension along *X*. *Y* and *Z* axis where providing security expert has choice of choosing any direction of *X*, *Y* and *Z* axis to propose their cryptosystem. The system can be more secure by combined effect of *X*, *Y* and *Z* axis. Further to get better resistance the sequence generated using particles of zig zag motion is injected to some chaotic maps for instance Basin chaotic map and Gingerbread man chaotic map to get highly randomized sequence. The output sequence of zig zag particles generated at first phase using Brownian motion is multiplied with one dimensional Basic chaotic map for better security. The process is further treated with bitwise XOR with sequence generated using Gingerbread man chaotic map. The sequence generated after third phase showed some exceptional randomness for security of multimedia data.

## 4. Proposed image encryption scheme

**Fig 2:**
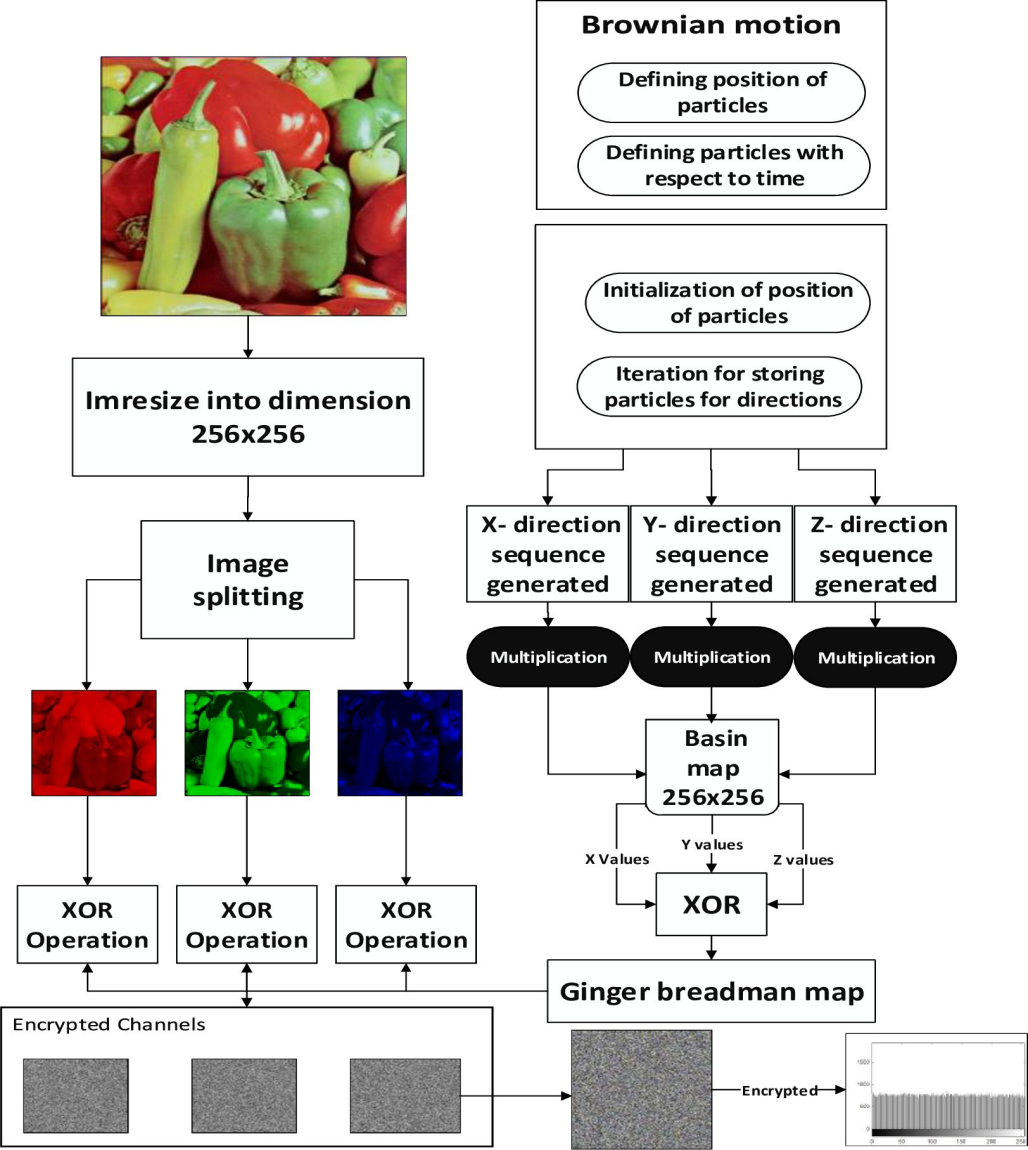
Flow chart of proposed algorithm for secure communication.

The main emphasis of this section is on the proposed encryption scheme algorithm. The major steps involved in suggested encryption scheme are given below (see Fig 2):

### 4.1 Steps involved in achieving secure algorithm

1 Read an image of size 512×512×3 in jpg format
2 Resizing of image from 512×512×3 to 256×256×3 dimension in same extension
3 Splitting of image into three layers of Red (R-layer), Green (G-Layer) and Blue (B-layer)
4 Generation of Brownian motion with initialization of position of particles with respect to time in seconds, number of particles assumed and number of impulses per change in track
5 Iteration of storing of position of particles for three different position along *X*, *Y*, and *Z* axis
6 Generation of two-dimensional Basin chaotic map having same length of 256×256
7 Multiplication of generated Basin chaotic map with three different position of particles generated in step 5
8 Generation of two-dimension gingerbread man chaotic map having same length of 256×256
9 Bitwise *XOR* operation between generated position in step 5 and generated gingerbread man chaotic map sequence having length of 256×256
10 The output in step 9 is again Bitwise *XOR* with the generated layers of Red, Green, Blue in step 3
12 Combining layer of images into 256×256×3 to get encrypted image

## 5. Security performance analysis and experimental results

In this section, we have taken standard images [51,67] to authenticate our proposed algorithm by utilizing the security benchmarks available in literature. The tests include are correlation coefficient, mean absolute error, means square error (MSE), peak signal to noise error (PSNR), information entropy, sensitivity analysis and NIST test for randomness respectively.

### 5.1 Histogram Analysis

**Fig 3:**
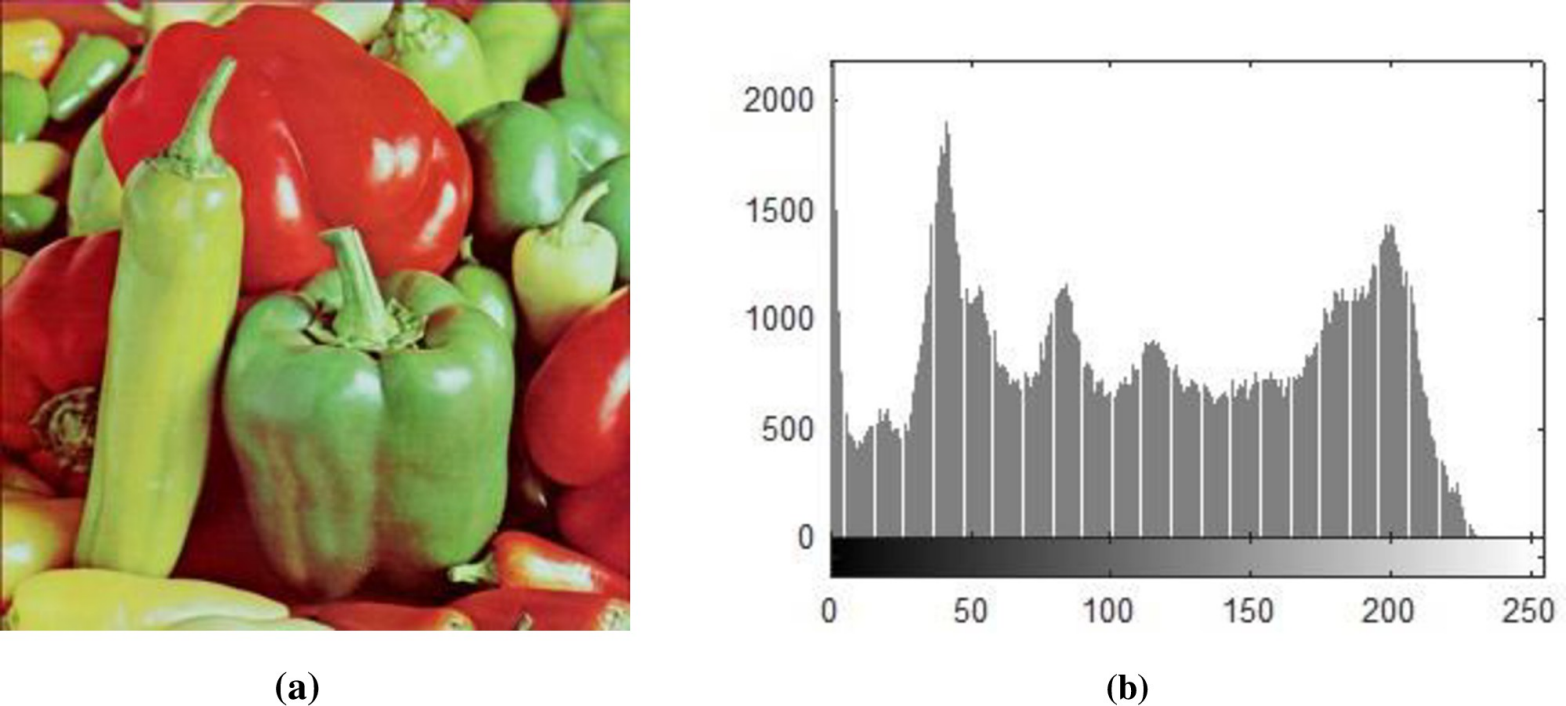
(a) Standard pepper image; (b) Histogram of pepper image.

**Fig 4:**
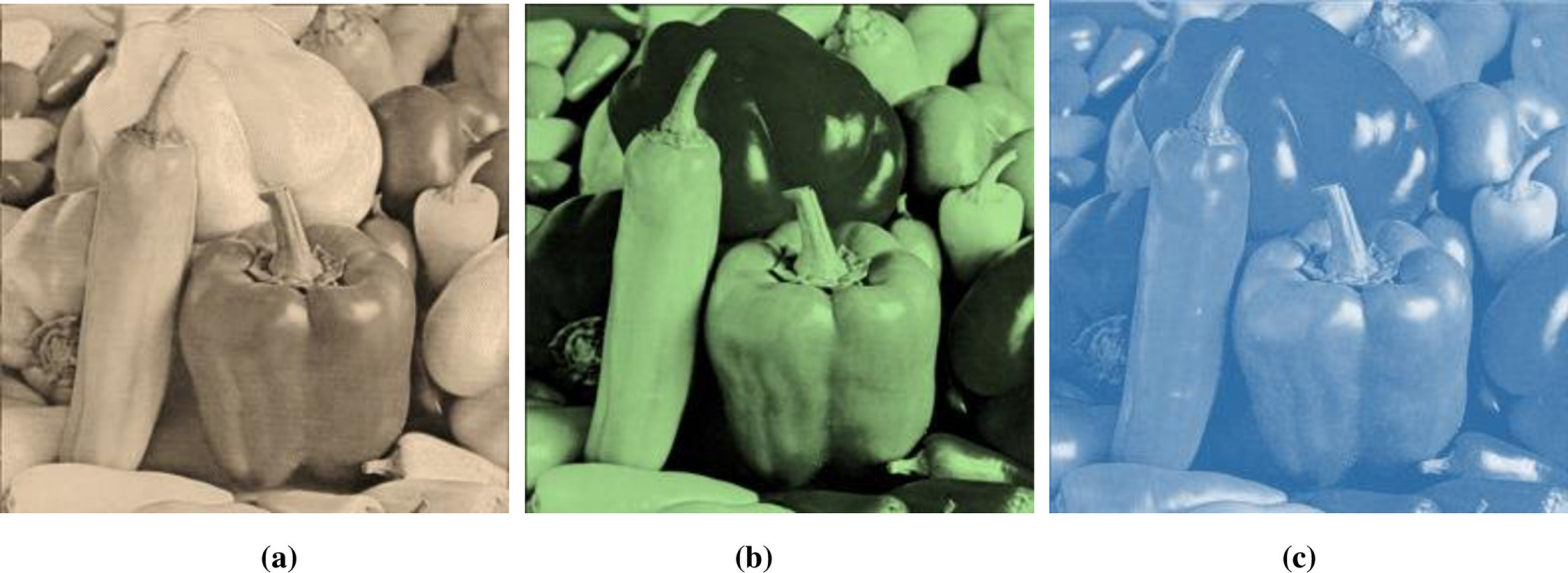
**(a)** R-layer of size 256×256; **(b)** G-layer of size 256×256; **(c)** B-layer of size 256×256.

**Fig 5:**
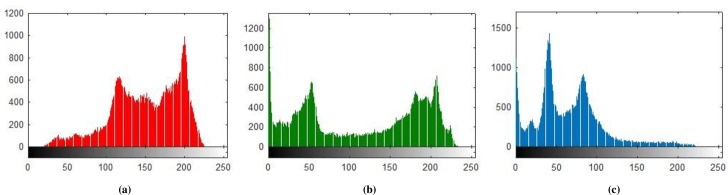
**(a)** R-layer Histogram; **(b)** G-layer Histogram; **(c)** B-layer Histogram.

**Fig 6:**
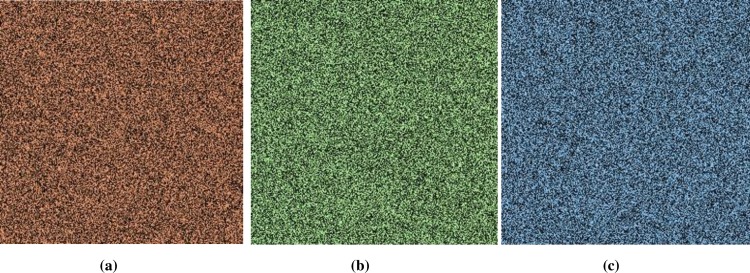
**(a)** X-direction R-layer; **(b)** X-direction G-layer; **(c)** X-direction B-layer.

**Fig 7:**
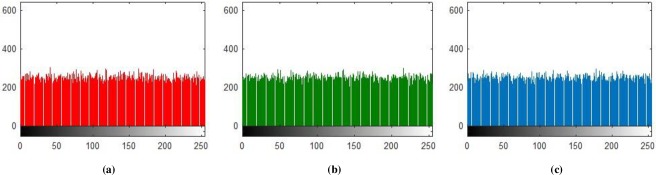
**(a)** X-direction R-layer Histogram; **(b)** X-direction G-layer Histogram; **(c)** X-direction B-layer Histogram.

**Fig 8:**
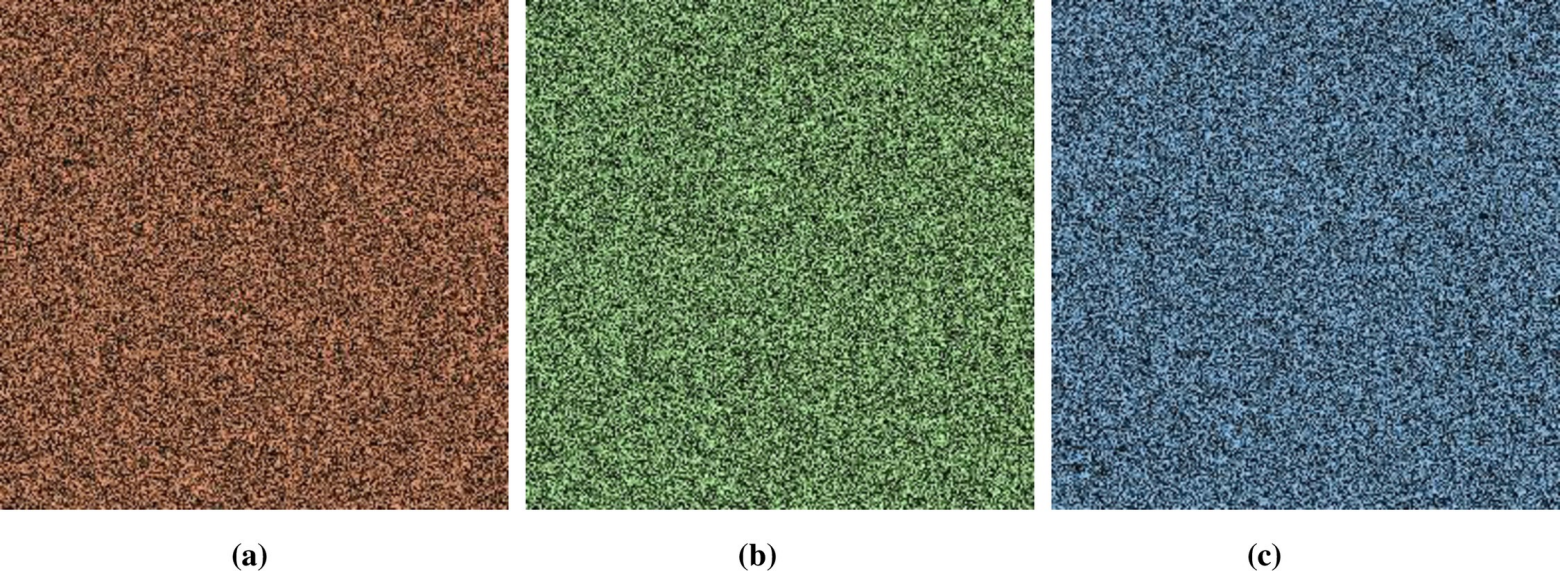
**(a)** Y-direction R-layer; **(b)** Y-direction G-layer; **(c)** Y-direction B-layer.

**Fig 9:**
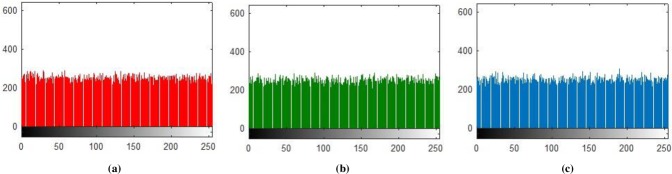
**(a)** Y-direction R-layer Histogram; **(b)** Y-direction G-layer Histogram; **(c)** Y-direction B-layer Histogram.

**Fig 10:**
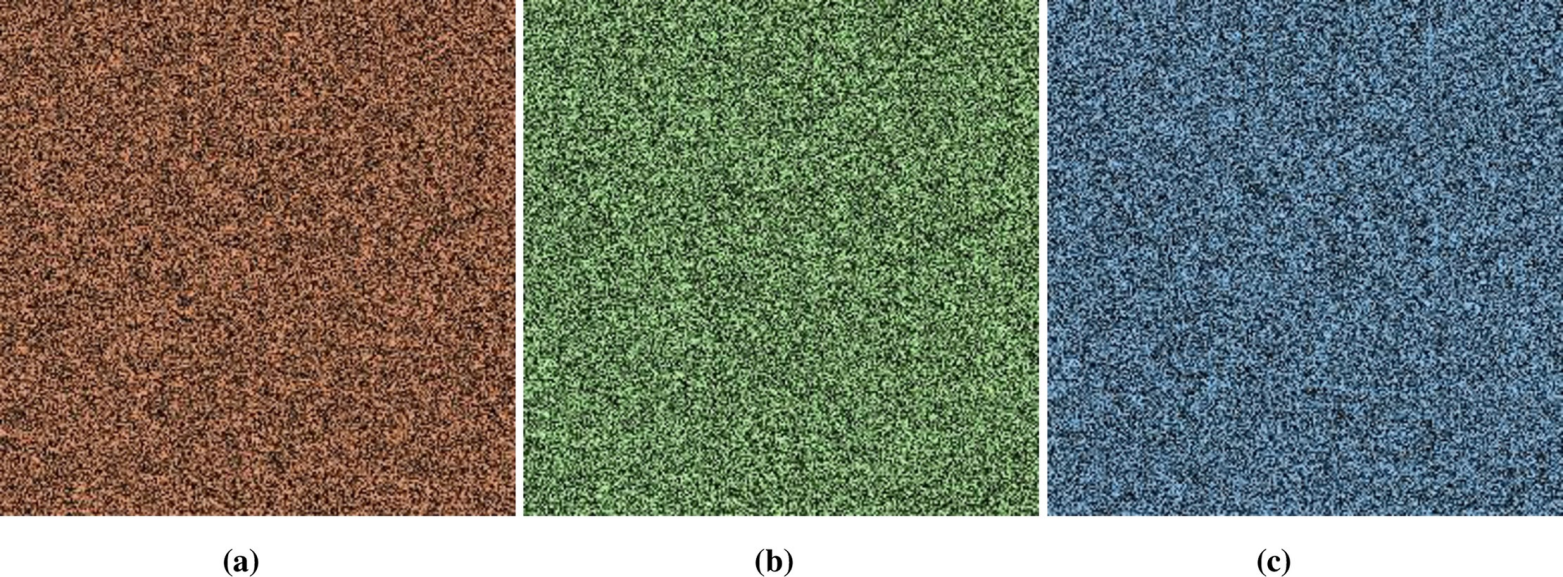
**(a)** Z-direction R-layer; **(b)** Z-direction G-layer; **(c)** Z-direction B-layer.

**Fig 11:**
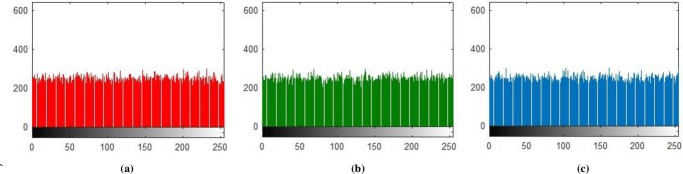
**(a)** Z-direction R-layer Histogram; **(b)** Z-direction G-layer Histogram; **(c)** Z-direction B-layer Histogram.

**Fig 12:**
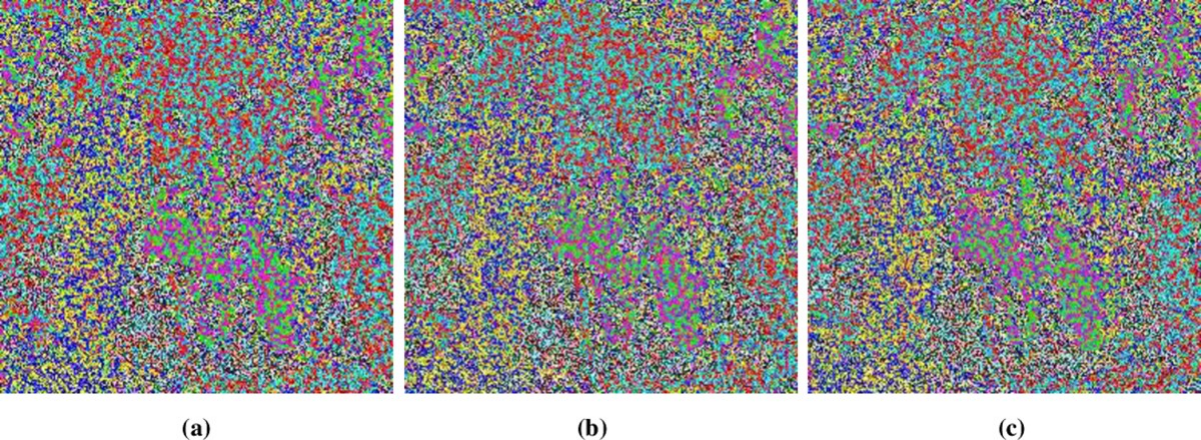
**(a)** X-direction combined-layers; **(b)** Y-direction combined-layers; **(c)** Z-direction combined-layers.

**Fig 13:**
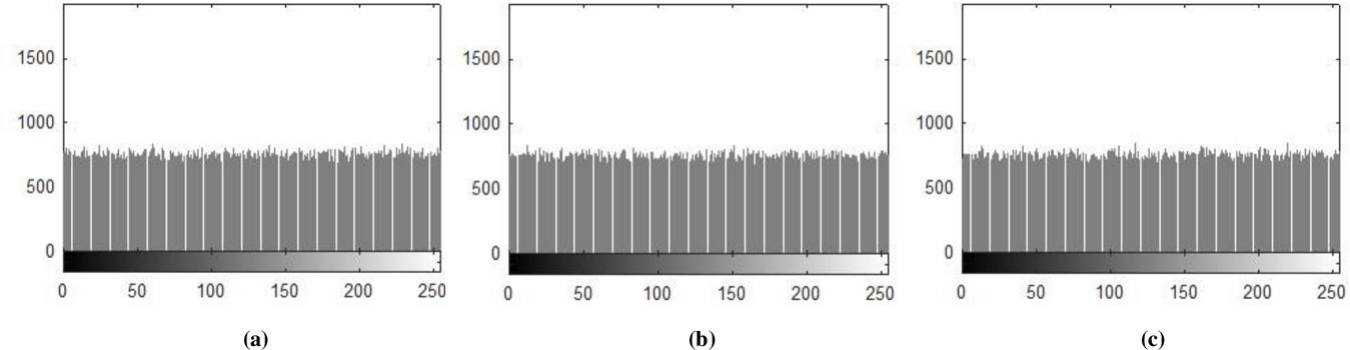
**(a)** X-direction combined-layers Histogram; **(b)** Y-direction combined-layers Histogram; **(c)** Z-direction combined-layers Histogram.

Histogram analysis is one of the momentous examination of pixels of image. Histogram must be different for plain and encrypted image. Plain image pixels are non-uniform and changes at each and every instant which reveals that the data is highly vulnerable to be attacked, while examining pixels of secure images where each pixel is well disciplined and have uniformly order having same length, size to one another. The well-disciplined and uniformity of pixels means that image is secure using proposed cryptosystem. We considered four images of 512×512×3 dimension and convert it into size of 256×256×3. The infrequent histogram and smooth continual is examined in Figs 3–13 respectively. Fig 3B show nature of non-uniformity as compare to succeeding Fig 13A–13C for three different directions along *X*, *Y* and *Z* axis where each pixel show resemblance to nearby pixel reveals that intruder will unable to decide that where maximum confidential information is available. Fig 4A–4C are layer wise images for pepper standard image having size 256×256×3 while the histogram for the layer wise figures are non-uniform reveals that the information can easily be take out on public network as shown in Fig 5A–5C. Looking into Figs 7, 9, 11 and 13, the Figs 7, 9 and 11 are layer wise different orientation of histogram where each pixel is equally distributed in the range of 0 to 256 and each pixel information is highly secure. Fig 13 is histogram of combined layers for three directions and show good pixels’ uniformity from start to end.

### 5.2 Correlation Coefficient

**Fig 14:**
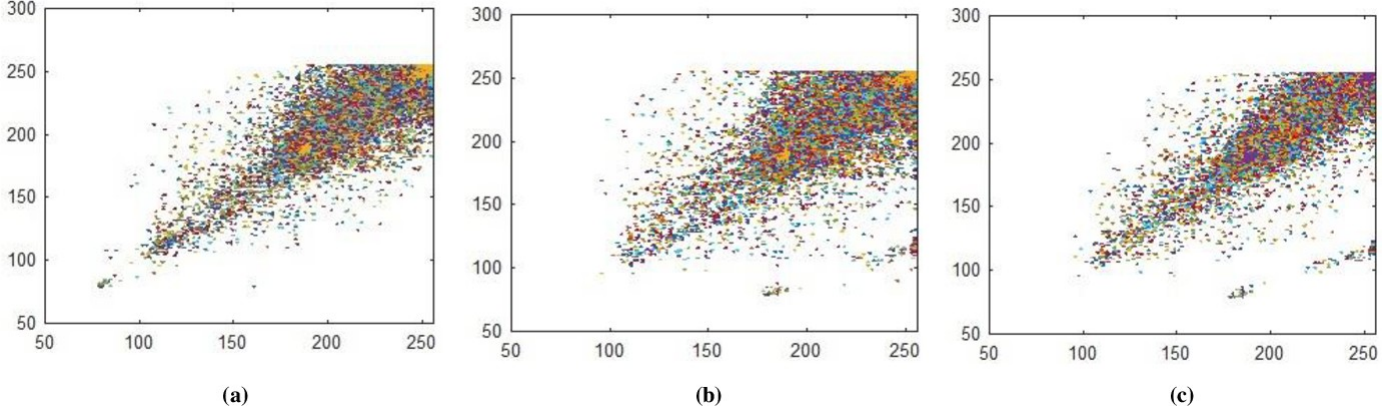
**(a)** Plain red layer horizontal correlation; **(b)** Plain red layer diagonal correlation; **(c)** Plain red-layer vertical correlation.

**Fig 15:**
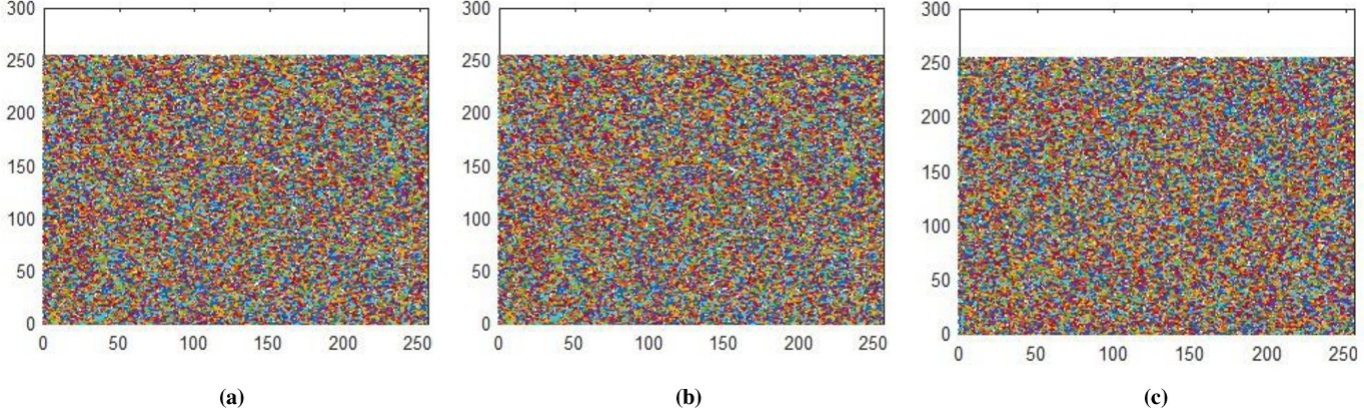
**(a)** Plain green layer horizontal correlation; **(b)** Plain green layer diagonal correlation; **(c)** Plain green layer vertical correlation.

**Fig 16:**
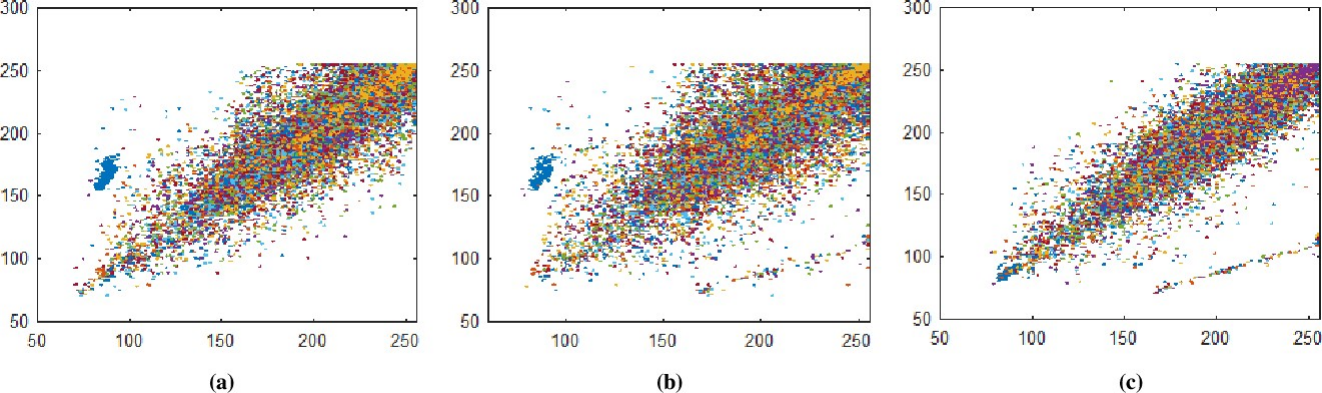
**(a)** Plain blue layer horizontal correlation; **(b)** Plain blue layer diagonal correlation; **(c)** Plain blue layer vertical correlation.

**Fig 17:**
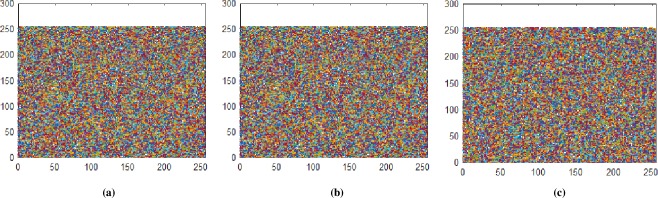
**(a)** Ciphered red layer horizontal correlation; **(b)** Ciphered red layer diagonal correlation; **(c)** Ciphered red layer vertical correlation.

**Fig 18:**
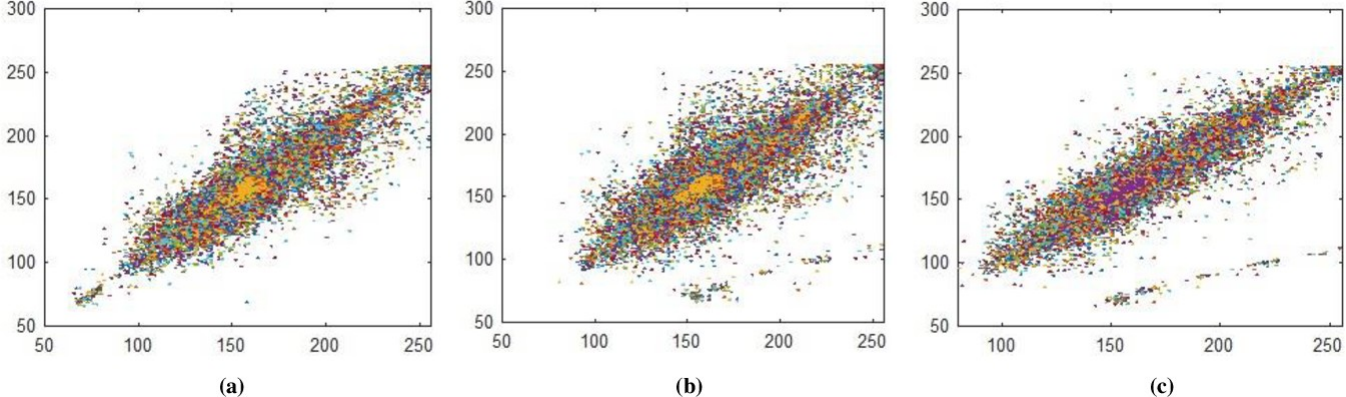
**(a)** Ciphered green layer horizontal correlation; **(b)** Ciphered green layer diagonal correlation; **(c)** Ciphered green layer vertical correlation.

**Fig 19:**
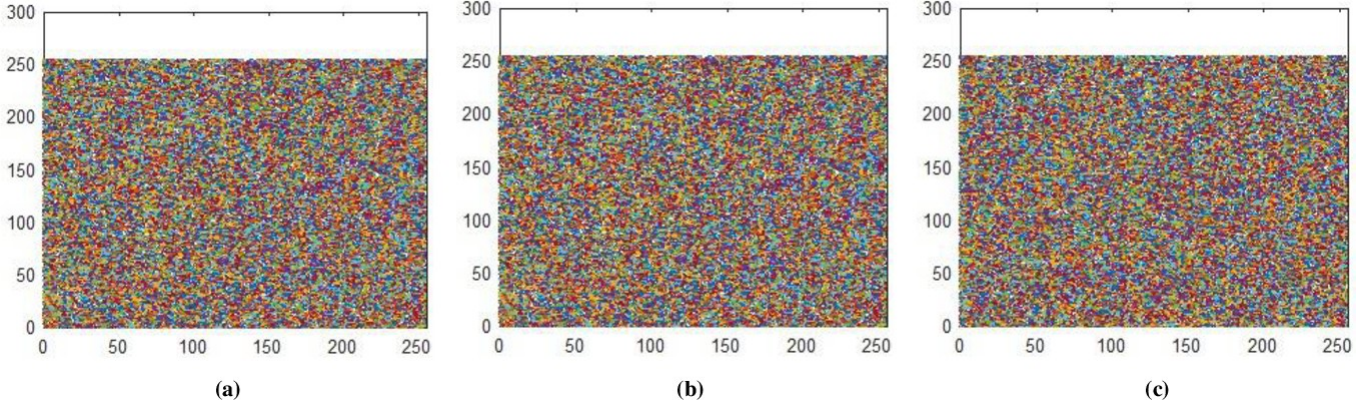
**(a)** Ciphered blue layer horizontal correlation; **(b)** Ciphered blue layer diagonal correlation; **(b)** Ciphered blue layer vertical correlation.

**Fig 20:**
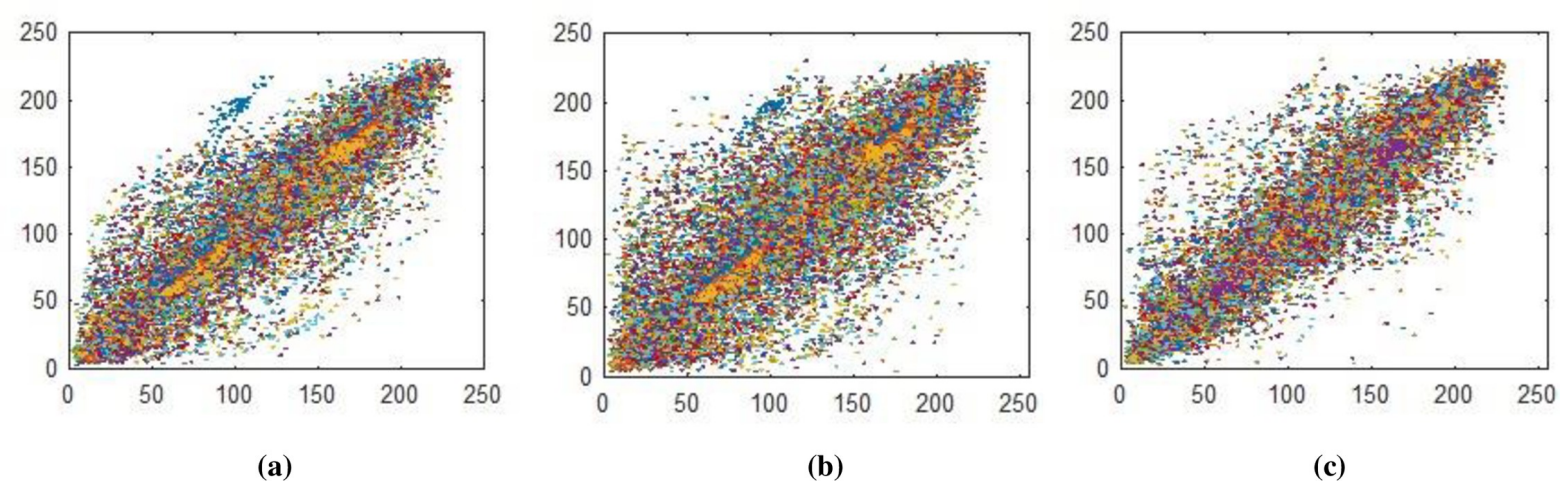
(a) Plain pepper image having size 256×256×3 horizontal correlation; (b) Plain pepper image having size 256×256×3 diagonal correlation; **(c)** Plain pepper image having size 256×256×3 vertical correlations.

**Fig 21:**
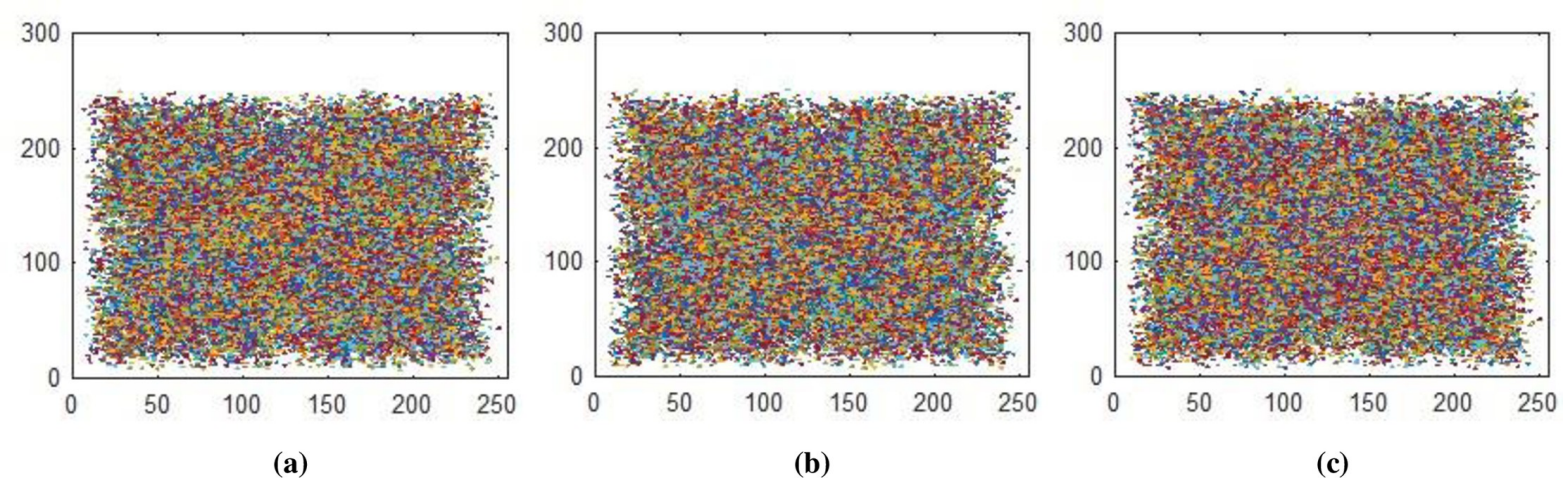
(a) Ciphered pepper image having size 256×256×3 horizontal correlation; **(b)** Ciphered pepper image having size 256×256×3 diagonal correlation; **(c)** Ciphered pepper image having size 256×256×3 vertical correlations.

Correlation coefficient analysis show resemblance of nearby image pixels along three different directions namely horizontal, vertical, and diagonal. For plain image the pixel’s value approaches to 1, while for encrypted image the value converges to 0. In Figs 14–16, all values are making diagonal line showing that values are highly correlated to each other. The below Eq (5) is use to find out pixel values at two different stages i.e. measuring pixels’ value when image is in the form of plain text and then finding pixels value when the image is in encrypted form. In proposed algorithm, we investigated pixels in three different directions for four types of test images along *X*-direction of Brownian motion. Figs 14–19 are layer wise correlation of red, green and blue channels having size of 256×256 whilst Figs 20 and 21 illustrates combined channels correlation having size of 256×256×3. Figs 14–16, are plain red, green, blue channels where each pixel is highly correlated to one another while Figs 17–19, where pixels are distributed in all direction in the range of 0 to 255 i.e. pixels gained behavior of dissimilarity after passing it through encryption algorithm. Fig 20 show highly resemblance of pixels in three directions while Fig 21 show pixels became scattered and pixels are no more similar. All analyzed tests are done for image having size of 256×256×3 and 256×256 respectively. Results for each direction of channel is shown in Tables 1–3. Table 1 has four images of 256×256 dimension while ‘*X*’ direction of Brownian motion is considered. The results are evaluated for plain and encrypted images. The average value of plain image for pepper is taken and evaluated its average results which are 0.9565, 0.9638, 0.9489 respectively. The value of plain image for pepper is nearly equal to ‘1’ show highly similarity. Same as, average values for cipher red, green and blue channels along ‘*X*’ direction are 0.0014, 0.0012, 0.0014 reveals that system is highly secure. The succeeding Table 2 with four standard channels of pepper, airplane, splash and tiffany having dimension of 256×256×3 with three distinct orientation of horizontal, vertical and diagonal is shown. The average value of plain for pepper image is 0.9573 and cipher value -0.0003 i.e. plain image value is nearly equal to one and cipher image is nearly equal to zero. In the very next Table 3 we have compared our proposed correlation values with some existing scheme values which validated our proposed system robustness. Correlation coefficient can be defined by using the following mathematical expression:

$$r=\frac{\sigma_{xy}}{\sigma_{x}\sigma_{y}},$$ (5)

where *σ*_*xy*_ is covariance and *σ*_*x*_, *σ*_*y*_ are standard deviations of random variables *x* and *y* respectively. The correlation coefficient for layer wise of Brownian motion into *X* direction is presented in Figs 14–21 respectively.

**Table 1:** Correlation coefficient for layer wise into three different directions.

		Plain values				Cipher values			
Image	Channel	H-D	D-D	V-D	A-V	H-D	D-D	V-D	A-V
Pepper	R-X	0.9646	0.9369	0.9680	0.9565	0.0011	0.0002	0.0030	0.0014
	**G-X**	0.9698	0.9466	0.9750	0.9638	-0.0016	0.0067	-0.0014	0.0012
	**B-X**	0.9570	0.9263	0.9636	0.9489	0.0018	0.0040	-0.0015	0.0014
**Airplane**	**R-X**	0.9389	0.8738	0.9239	0.9122	-0.0030	0.0012	-0.0056	-0.0074
	**G-X**	0.9309	0.8814	0.9343	0.9155	0.0006	-0.0009	-0.0053	-0.0018
	**B-X**	0.9503	0.8800	0.9089	0.9130	-0.0094	-0.0034	-0.0016	-0.0048
**Splash**	**R-X**	0.9870	0.9824	0.9947	0.9880	-0.0009	0.0001	-0.0012	-0.0020
	**G-X**	0.9688	0.9519	0.9787	0.9664	-0.0044	0.0031	-0.0023	-0.0012
	**B-X**	0.9706	0.9407	0.9660	0.9591	0.0005	0.0011	-0.0003	0.0004
**Tiffany**	**R-X**	0.9542	0.9129	0.9486	0.9385	-0.0038	0.0004	-0.0045	-0.0026
	**G-X**	0.9262	0.8812	0.9467	0.9180	-0.0016	0.0027	-0.0008	0.0001
	**B-X**	0.9348	0.8850	0.9376	0.9191	-0.0005	0.0045	-0.0024	0.0005

**R-X:** Red layer in X direction; **G-X:** Green layer in X direction; **B-X:** Blue layer in X direction; **H-D**: Horizontal direction; **D-D**: Diagonal direction; **V-D**: Vertical direction; **A-V**: Average value.

**Table 2:** Correlation coefficient values for several standard test images.

		Plain values				Encrypted values			
Images	Dimension	H	D	V	A V	H	D	V	A V
**Pepper**	256×256	0.9638	0.9371	0.9711	0.9573	-0.0014	0.0043	-0.0032	-0.0003
**Airplane**	256×256	0.9346	0.8819	0.9301	0.9155	0.0051	0.0047	-0.0011	0.0029
**Splash**	256×256	0.9724	0.9604	0.9843	0.9723	-0.0073	-0.0031	0.0066	-0.0012
**Tiffany**	256×256	0.9542	0.9129	0.9486	0.9385	0.0020	-0.0027	0.0065	0.0019

**H:** Horizontal; **D:** Diagonal; **V:** Vertical; **A V:** Average values.

**Table 3:** Comparison of correlation coefficient values with existing techniques.

Algorithms	H-D	D-D	V-D
Proposed Plain image	0.9638	0.9371	0.9711
Proposed cipher image	-0.0014	0.0043	-0.0032
Ref. [32]	0.0022	0.0001	-0.0017
Ref. [33]	0.1257	0.0581	0.0504
Ref. [34]	0.0681	0.0845	-
Ref. [35]	0.0024	0.0580	0.0170
Ref. [36]	0.0008	0.0008	0.0005
Ref. [37]	0.0036	0.0023	0.0039
Ref. [38]	0.0044	0.0034	0.0020
Ref. [39]	0.0012	0.0026	0.0021
Ref. [40]	0.0024	0.0012	0.0016
Ref. [41]	0.0046	0.0040	0.0017
Ref. [42]	0.0072	0.0058	0.0031
Ref. [43]	0.0214	0.0465	-0.0090
Ref. [44]	0.0845	-	0.0681
Ref. [45]	0.0965	0.0362	-0.0581
Ref. [46]	0.1257	0.0226	0.0581

**H-D:** Horizontal direction; **D-D:** Diagonal direction; V-D: Vertical direction.

### 5.3 Mean square error

To compute accuracy of system, mean square error is to be measure. Mean square error can be computed from plain and cipher image using Eq 8 as shown:

$$MSE=\frac{1}{M\times N}\sum_{i=1}^{M}\sum_{j=1}^{N}\left(O_{\left(i,j\right)}-E_{\left(i,j\right)}\right).$$ (6)

Bigger mean square error (MSE) value show the system is more secure against differential attacks. The calculated mean square error values are shown in Table 4.

**Table 4:** MSE and PSNR tests for various standard images.

		Technique types	
Images	Dimension	MSE	PSNR
Peppers	256×256	10925.35	7.74
	256×256	10965.37	7.73
	256×256	10939.40	7.74
Airplane	256×256	9867.87	8.22
	256×256	10565.08	7.93
	256×256	10375.41	8.00
Splash	256×256	11405.02	7.59
	256×256	12294.93	7.27
	256×256	9845.95	8.23
Tiffany	256×256	17631.99	5.70
	256×256	13063.02	7.00
	256×256	7323.95	9.52

### 5.4 Peak signal to noise ratio

To evaluate image quality of cipher image, peak to signal noise ratio (PSNR) is to be measure which can be illustrated using Eq 9 as shown:

$$PSNR=10\log_{2}\frac{I_{\max}^{2}}{MSE}.$$ (7)

The peak signal to noise ratio (PSNR) must be minimum to ensure good security. The two criterions are conflicting in nature. Mean square error must be approaching to maximum and peak to signal noise ratio must be approaching to minimum to complete security requirements that needed to design algorithm. PSNR calculated values using proposed algorithm are shown in Table 4. We evaluated tests for four standard images of pepper, airplane, splash and tiffany of having size 256×256×3. The average value for MSE that must be near to 10000 while for better peak to signal noise ratio the average value must be near to 8.50. Looking into Tables 4 and 5 for pepper as a test image for three respective channels. Average value of MSE for all three channels are 10943.37 while average PSNR value is almost equal to 7.74. The very next standard image is airplane having same length of 256×256 calculates MSE value which is greater than 10000 and PSNR value less than 8.50. The succeeding image splash having average MSE value with 11181.96 is exceptional after tiffany image having value of 12672.98 for the same size with average PSNR is less than 8.50. The final image tiffany having same size of 256×256×3 gained MSE output result equal to 12672.98 with peak to signal noise ratio having 7.40. PSNR and MSE average for different four standard images authenticated robustness of proposed cryptosystem. Table 6 show comparison of MSE and PSNR with existing algorithms.

**Table 5:** Average MSE and PSNR values for certain test images.

		Projected technique	
Images	Dimensions	Avg. MSE	Avg. PSNR
Pepper	256×256×3	10943.37	7.74
Airplane	256×256×3	10269.45	8.05
Splash	256×256×3	11181.96	7.69
Tiffany	256×256×3	12672.98	7.40

**Table 6:** Comparison of existing MSE values with proposed cryptosystem output values.

Preexisting algorithms	Dimensions	Compared MSE Values
Ref. [47]	256×256×3	8369
Ref. [48]	256×256×3	5800
Ref. [49]	256×256×3	3300
Ref. [50]	256×256×3	8760
AES	256×256×3	4600
AES-CBC	256×256×3	4637
AES-Counter	256×256×3	4938
AES-Feedback	256×256×3	4577
AES-Stream	256×256×3	4911
Proposed	256×256×3	10943

### 5.5 Mean Absolute Error

Mean absolute error is criterion use to examine security of the system. Higher the value of mean absolute error, the stronger the system is designed. Suppose two images, the one plain image '*P*_*i*,*j*_' and another encrypted image '*E*_*i*,*j*_' be grey level of pixels at *i*^*th*^ row and *j*^*th*^ column having *M*×*N* size of plain and cipher image respectively. The Mean absolute error between two images of plain and encrypted images can be calculated using simple equation:

$$MAE=\frac{1}{M\times N}\sum_{i=0}^{M-1}\sum_{j=0}^{N-1}\left|E_{i,j}-P_{i,j}\right|$$ (8)

By investigating Table 7 for mean absolute error values for four standard test images having same length of 256×256×3. The average value for mean absolute error is approximately equal to 70 ~ 75. The larger the value is achieved; the more reliable and secure cryptosystem is designed. In Table 7 values for all four standard test images have shown. In the same table we compared the proposed cryptosystem values with already existing algorithm for same standard images having same size.

**Table 7:** MAE values for certain standard images and its comparison.

Image	Dimension	MAE value	Ref. [51]	Ref. [66]
Pepper	256×256	86	74	74
Airplane	256×256	101	74	
Splash	256×256	117	-	76
Tiffany	256×256	161	76	94

### 5.6 Information Entropy

Randomness test theory was put forward by Shannon in 1949 and estimated randomness and unpredictability of information [44]. Information entropy analysis is one of the familiar methods of finding randomness of secure sequence. The ideal value for maximum entropy is always 8 for 8-bit system. We calculated entropy against each layer for different standard test images with different dimensions. The value examined using proposed algorithm is 7.999 which is very close to ideal value of 8 which validated our proposed cryptosystem and ensured its high security against any linear and differential attack. The comparison of computed layer as well combined form is shown in Tables 8–11 respectively. Information entropy can be computed using equation:

$$H(m)=\sum_{i=0}^{2^{K}-1}p(m_{i})\log_{b}(1/p(m_{i})),$$ (9)

where *p*(*m*_*i*_) is probability of massages ‘*m*’, 2^*K*^ means all possible outcomes where ‘*K* ’is number of bits included for each massage and ‘log_*b*_’ in equation is logarithm with logarithmic base. Looking into Tables 8–11. Table 8 are calculated values of entropy for different layers having length of 256×256×3 and 512×512×3 image with channel wise having length of 256×256 and 512×512 respectively. The ideal entropy value is always 8 for 8-bit system. The evaluated values of channel wise pepper image have some exceptional outputs of 7.997, 7.997, 7996 which approximates to ideal value, further the values are evaluated for number of standard test images for different channels are shown in Table 8. In Table 9 we compared our evaluated value of proposed cryptosystem with already existing values. The proposed value of 7.999 is almost equal to ideal value of maximum randomness of 8 for 8-bit system which validated robustness of our proposed cryptosystem. In Table 10 we compared results of proposed algorithm of 256×256×3 with several existing algorithms. In Table 11 images having size of 256×256×3 and 512×512×3 is shown. This concluded that system show some extra behavior of robustness to be breakdown against linear and differential passwords.

**Table 8:** Information entropy analysis for certain standard images of dimension 256×256 and 512×512.

Image	Channels	Dimension	Evaluated entropy values
Peppers	***RC***	256×256	7.997
	***GC***	256×256	7.997
	***BC***	256×256	7.996
	***RC***	512×512	7.999
	***GC***	512×512	7.999
	***BC***	512×512	7.999
Airplane	***RC***	256×256	7.997
	***GC***	256×256	7.997
	***BC***	256×256	7.997
	***RC***	512×512	7.999
	***GC***	512×512	7.999
	***BC***	512×512	7.999
Splash	***RC***	256×256	7.997
	***GC***	256×256	7.997
	***BC***	256×256	7.997
	***RC***	512×512	7.999
	***GC***	512×512	7.999
	***GC***	512×512	7.999
Tiffany	***RC***	256×256	7.996
	***GC***	256×256	7.997
	***BC***	256×256	7.997
	***RC***	512×512	7.999
	***GC***	512×512	7.999
	***BC***	512×512	7.999

**RC:** Red channel; **GC:** Green channel; **BC:** Blue channel

**Table 9:** Comparison of proposed image entropy value with existing algorithm entropy.

Algorithms	Dimension	Compared values
Proposed image	256×256×3	7.9992
Ref [52]	256×256×3	7.9993
Ref [53]	256×256×3	7.9990
Ref [54]	256×256×3	7.9974
Ref [55]	256×256×3	7.9971
Ref [56]	256×256×3	7.9970

**Table 10:** Comparison of proposed layer wise entropy values with existing values of certain algorithms.

Algorithms	Dimension	Red	Green	Blue
Rhouma et al. [44]	256×256×3	7.9732	7.9750	7.9715
Hongjun et al. [45]	256×256×3	7.9851	7.9852	7.9832
Huang et al. [46]	256×256×3	7.8501	7.9028	7.5582
Proposed	256×256×3	7.997	7.997	7.996

**Table 11:** Entropy values for certain standard images with dimension of 256×256×3 and 512×512×3.

Images	Dimension	Projected technique
Pepper	256×256	7.999
	512×512	7.999
Airplane	256×256	7.999
	512×512	7.999
Splash	256×256	7.999
	512×512	7.999
Tiffany	256×256	7.999
	512×512	7.999

### 5.7 Sensitivity analysis

The number of changing pixel rate (NPCR) and the unified averaged changed intensity (UACI) are two standardized tests use to examine plain image sensitivity against external differential attack. This test elaborates one effect on another i.e. making very small change in plain image will cause immense difference in the corresponding encrypted images. Higher the value of NPCR is achieved, the more effective cryptosystem is designed and will highly oppose differential and linear attacks. The ideal value for NPCR is always 100. The values calculated using proposed cryptosystem is around to 99.64 which is too close to 100 shows the system is strong enough against attack. UACI test calculates average intensity change between plain and cipher image. The value must be near to 33. The examined values for the proposed image of pepper is around to 33 which is higher than expected value ensure robustness of cryptosystem.

#### 5.7.1 Number of changing pixel rate

In this test we analyzed difference in cipher images with change in single pixel of source image. The test evaluated values are shown in Table 12. We represented first cipher image as *E*_1(*i*,*j*)_ and second cipher *E*_2(*i*,*j*)_ image as respectively.

$$NPCR=\frac{\sum_{i,j}F(i,j)}{W\times H}\times 100\%,$$ (10)

where *F*_*i*,*j*_ = 0 for *E*_1(*i*,*j*)_ = *E*_2(*i*,*j*)_ and *F*_*i*,*j*_ = 1 for *E*_1(*i*,*j*)_ ≠ *E*_2(*i*,*j*)_.

**Table 12:** Layer wise NPCR and UACI values of standard images.

			Projected technique	
Image	Layer type	Dimension	NPCR	UACI
Pepper	R-L	256×256	99.64	33.49
	G-L	256×256	99.64	33.56
	B-L	256×256	99.64	33.50
Airplane	R-L	256×256	99.65	32.48
	G-L	256×256	99.65	32.48
	B-L	256×256	99.65	32.48
Splash	R-L	256×256	99.67	33.87
	G-L	256×256	99.67	33.87
	B-L	256×256	99.67	33.87
Tiffany	R-L	256×256	99.62	36.11
	G-L	256×256	99.62	36.11
	B-L	256×256	99.62	36.11

#### 5.7.2 Unified averaged changed intensity

It is one of the important examinations for ensuring whether the proposed system is robust against differential attack or not. This examination is based on intensity of difference between two images. UACI can be computed using formula given:

$$UACI=\frac{1}{W\times H}\sum_{i,j}\left[\frac{E_{1(i,j)}-E_{2(i,j)}}{255}\right]\times 100\%.$$ (11)

Table 12 show UACI test is applied for different test images having size of 256×256×3 and investigated its result for layer wise respectively. We evaluated results for the same standard images with dimension of 256×256 in layer wise. The standard value of NPCR is approximately equal to 9.60 whilst the achieved values are more than 9.60 ensure proposed algorithm is secure. The UACI average values of 33.51, 32.48, 33.87 and 36.11 also confirmed security of proposed algorithm is remarkable (see Table 13).

**Table 13:** Comparison of Proposed algorithms for NPCR and UACI test with various existing algorithms.

	Avg. NPCR comparison	Avg. UACI comparison
Proposed algorithm	99.64	33.51
Ref [57]	99.52	26.79
Ref [58]	99.58	33.37
Ref [59]	99.59	17.60
Ref [60]	99.60	33.23
Ref [61]	99.60	28.13
Ref [62]	99.55	33.40
Ref [63]	99.59	33.46

### 5.8 Time complexity analysis

Time complexity is one of the most important analysis use to find out efficiency of certain cryptographic algorithms. This test describes actual time taken in execution of encryption using proposed algorithm. The proposed system is very much efficient then already existing schemes. The efficiency of system depends upon computational cost and resources which must be minimum to ensure that system is much efficient then already existing schemes. We investigated our proposed encryption algorithm using windows 10 pro operating system with system specification that includes CPU core^™^ i3 3227U, 1.9 GHZ with 8 GB ram and MATLAB 2017(a) version. Table. 14 demonstrates the time taken during encryption of proposed scheme. The test is done for several standard images and compared to already existing cryptosystem encryption time complexity. The encryption and decryption time are almost same for unique cryptosystem. The projected technique in Table. 14 show that the proposed hybrid system of Brownian movement of particles with certain chaotic dynamical system is highly efficient. This test validated our proposed cryptosystem and ensured the efficiency of system.

**Table 14:** Time complexity analysis for certain images.

Images	Proposed schemes	Ref. [68]	Ref. [69]
Pepper	2.17	2.76	3.68
Lena	2.14	2.25	3.23
Splash	2.17	-	-
Airplane	2.10	-	-
Sailboat	2.13	2.66	3.55
Baboon	2.17	2.55	3.53

### 5.9 Investigation of NIST test

NIST test examination is one the most notable approved standard use to find randomness. This test involves number of autonomous statistical tests. We have investigated NIST tests for our proposed encryption algorithm in order to authenticate the randomness. The probabilities of passing each test must be greater than 0.01. In case of our anticipated scheme, the number values for NIST are all greater than 0.01 which qualify the suggested scheme for true random cipher (See Table 15).

**Table 15:** NIST test analysis for different layers of encrypted image.

			Color components of image layers			
Test names			Red	Green	Blue	Remarks
Frequency			0.37592	0.2059	0.24198	Success
Block Frequency			0.38274	0.68179	0.47892	Success
Run (m = 10000)			0.11664	0.86933	0.48024	Success
Long runs of ones			0.7127	0.7127	0.67514	Success
Rank			0.29191	0.29191	0.29191	Success
Spectral DFT			0.77167	0.01364	0.46816	Success
No overlapping			1.00000	0.99981	0.99999	Success
Overlapping			0.85988	0.85988	0.85988	Success
Universal			0.99277	0.9835	0.99631	Success
Serial 1		*p values*	0.14236	0.05063	0.2845	Success
Serial 2		*p values*	0.75273	0.04634	0.53489	Success
Approx. Entropy			0.77216	0.06849	0.62349	Success
Cumulative sum forward			0.41149	0.35256	0.049371	Success
Cumulative sum reverse			0.77506	0.91758	1.5885	Success
		*X = -4*	0.22336	0.02894	0.59869	Success
		*X = -3*	0.00065	0.00038	0.78206	Success
		*X = -2*	0.00148	0.52378	0.61767	Success
		*X = -1*	0.28867	0.6131	0.7699	Success
Random excursions		*X = 1*	0.81044	0.58412	0.89735	Success
		*X = 2*	0.21918	0.73098	0.89552	Success
		*X = 3*	0.8282	0.75335	0.81792	Success
		*X = 4*	0.08361	0.8704	0.51758	Success
		*X = -4*	0.87737	0.20771	0.32658	Success
		*X = -3*	0.58933	0.13604	0.24574	Success
		*X = -2*	0.63735	0.21096	0.34897	Success
Random excursion variants		*X = -1*	1.00000	0.13361	0.7456	Success
		*X = 1*	0.83826	0.18242	0.87113	Success
		*X = 2*	0.81366	0.28984	0.92538	Success
		*X = 3*	0.64808	0.23304	0.77167	Success
		*X = 4*	0.58915	0.25684	0.58107	Success

### 5.10 Software and system specifications

In this section we conducted tests for several standard test images having size of 256×256×3 and layer wise having size of 256×256 and OS of CPU core^™^ i3 3227U, 1.9 GHZ with 8 GB ram and MATLAB 2017(a) version.

## 6 Conclusion

We have proposed a digital confidentiality preserving scheme which is based on Brownian motion and chaotic iterative maps. The suggested scheme is authenticated against various standard benchmarks available in literature. Our proposed scheme is quite competent of providing secrecy to digital contents and prevent eavesdropper to steals the secret information. The proposed algorithm is designed for security of images though it can be extended to the encryption of some other types of data such as audio and video information. The system designed can be implemented for real time communication.
