# Expression of Concern: A novel image encryption technique using hybrid method of discrete dynamical chaotic maps and Brownian motion

**DOI:** 10.1371/journal.pone.0339232

**Published:** 2025-12-19

**Authors:** The PLOS One Editors

After this article [1] was published, the following concerns were noted:

- The articles cited as Reference 3, 9 and 51 were retracted after [1] was published due to concerns about potential manipulation of the publication process.
- The article exhibits abnormal citation patterns (e.g. the citation of references [17-70] in support of a single statement).

In light of the cumulative issues, the *PLOS One* Editors issue this Expression of Concern. Readers are advised to interpret the article [1] with caution.
